# Lipocalin 2 Influences Bone and Muscle Phenotype in the *MDX* Mouse Model of Duchenne Muscular Dystrophy

**DOI:** 10.3390/ijms23020958

**Published:** 2022-01-16

**Authors:** Marco Ponzetti, Argia Ucci, Antonio Maurizi, Luca Giacchi, Anna Teti, Nadia Rucci

**Affiliations:** Department of Biotechnological and Applied Clinical Sciences, University of L’Aquila, 67100 L’Aquila, Italy; marco.ponzetti@graduate.univaq.it (M.P.); argia.ucci@graduate.univaq.it (A.U.); antonio.maurizi@guest.univaq.it (A.M.); luca.giacchi@student.univaq.it (L.G.); annamaria.teti@univaq.it (A.T.)

**Keywords:** Lipocalin 2, Duchenne Muscular Dystrophy, bone, muscle, osteoporosis, inflammation

## Abstract

Lipocalin 2 (Lcn2) is an adipokine involved in bone and energy metabolism. Its serum levels correlate with bone mechanical unloading and inflammation, two conditions representing hallmarks of Duchenne Muscular Dystrophy (DMD). Therefore, we investigated the role of Lcn2 in bone loss induced by muscle failure in the *MDX* mouse model of DMD. We found increased Lcn2 serum levels in *MDX* mice at 1, 3, 6, and 12 months of age. Consistently, *Lcn2* mRNA was higher in *MDX* versus WT muscles. Immunohistochemistry showed Lcn2 expression in mononuclear cells between muscle fibres and in muscle fibres, thus confirming the gene expression results. We then ablated *Lcn2* in *MDX* mice, breeding them with *Lcn2^−/−^* mice (*MDXxLcn2^−/−^*), resulting in a higher percentage of trabecular volume/total tissue volume compared to *MDX* mice, likely due to reduced bone resorption. Moreover, *MDXxLcn2^−/−^* mice presented with higher grip strength, increased intact muscle fibres, and reduced serum creatine kinase levels compared to *MDX*. Consistently, blocking Lcn2 by treating 2-month-old *MDX* mice with an anti-Lcn2 monoclonal antibody (Lcn2Ab) increased trabecular volume, while reducing osteoclast surface/bone surface compared to *MDX* mice treated with irrelevant IgG. Grip force was also increased, and diaphragm fibrosis was reduced by the Lcn2Ab. These results suggest that Lcn2 could be a possible therapeutic target to treat DMD-induced bone loss.

## 1. Introduction

Lipocalin 2 (Lcn2) is a multifunctional protein involved in inflammation [1], acute kidney injury [2], and bone [3,4,5,6] and muscle [7,8,9] pathophysiology. Notably, Lcn2 emerges as a complex player in bone biology, being detrimental for bone health when upregulated in hindlimb suspension and head-down tilt bed rest in mice and humans, respectively [4], but causing osteopenia in basal conditions when absent [6]. With regard to its role in muscle physiology, it has recently been proposed that removing Lcn2 impairs satellite cell proliferation in middle-age mice (6-month-old), eventually leading to reduced acute muscle damage repair [8]. Furthermore, omics analysis performed on the *longissimus dorsi* (LD) muscle explanted from mice subjected to space flight shows upregulation of Lcn2 compared to earth gravity control [7]. Furthermore, several reports claim that Lcn2 is a negative determinant of myocardial health [10,11,12,13]. Finally, we recently demonstrated that Lcn2 is increased following acute high-intensity exercise in humans and that it might be detrimental for muscle differentiation. In contrast, genetic ablation of Lcn2 in mice does not negatively affect muscle physiology [9]. Taken together, these findings show that Lcn2 is a complex molecule, playing different roles in different contexts.

Duchenne Muscular Dystrophy (DMD) is a rare X-linked genetic disorder, caused by mutations in the dystrophin gene. Its incidence is the highest known for a rare genetic disease, affecting up to 1/4000 males born live [14]. Dystrophin has a key structural role in the Dystrophin-Associated Glycoprotein Complex (DAGC), eventually promoting the connection between actin fibres within the sarcoplasm and the extracellular matrix. A lack of dystrophin causes DAGC loss-of-function, with consequent damage of the myofibres following muscle contraction. Muscle damage also increases calcium influx in the myofibre, exacerbating the contraction and worsening the damage [15]. Muscle necrosis then contributes to the establishment of a chronic pro-inflammatory milieu that causes further myofibre death [16].

A key phenomenon worsening the quality of life of DMD patients is the bone mechanical unloading caused by muscle atrophy, which eventually leads to bone loss and disuse osteoporosis, dramatically increasing the risk of fractures [17,18]. This phenomenon of disuse-induced bone loss is also frequent in other unloaded subjects, such as tetraplegic/paraplegic patients and astronauts [19,20]. The increase of fracture risk in DMD is due not only to the mechanical unloading of bone but also to the pro-inflammatory cascade induced by the disease, which exacerbates osteoclast activity and impairs osteoblast function [21]. Moreover, glucocorticoids, representing the standard of care for DMD patients, further increase bone loss, stimulating osteoclasts and inhibiting osteoblasts [22,23,24].

In this study, we aimed at investigating the role of Lcn2 as a determinant and potential therapeutic target in DMD-induced bone loss, using the *MDX* mouse model of this disease. We found that Lcn2 concentration is increased in sera and muscle of *MDX* mice compared to wild-type (WT) mice and correlates with the serum levels of pro-inflammatory cytokines. Moreover, the genetic ablation of Lcn2 in mice or the neutralisation of its function by an Lcn2 blocking antibody, rescue the bone volume in *MDX* mice at different ages, with a beneficial effect on muscle health.

## 2. Results

### 2.1. Lcn2 Levels Are Increased in MDX Mice and Correlate with Il1b and Tnfa Levels

**Figure 1:**
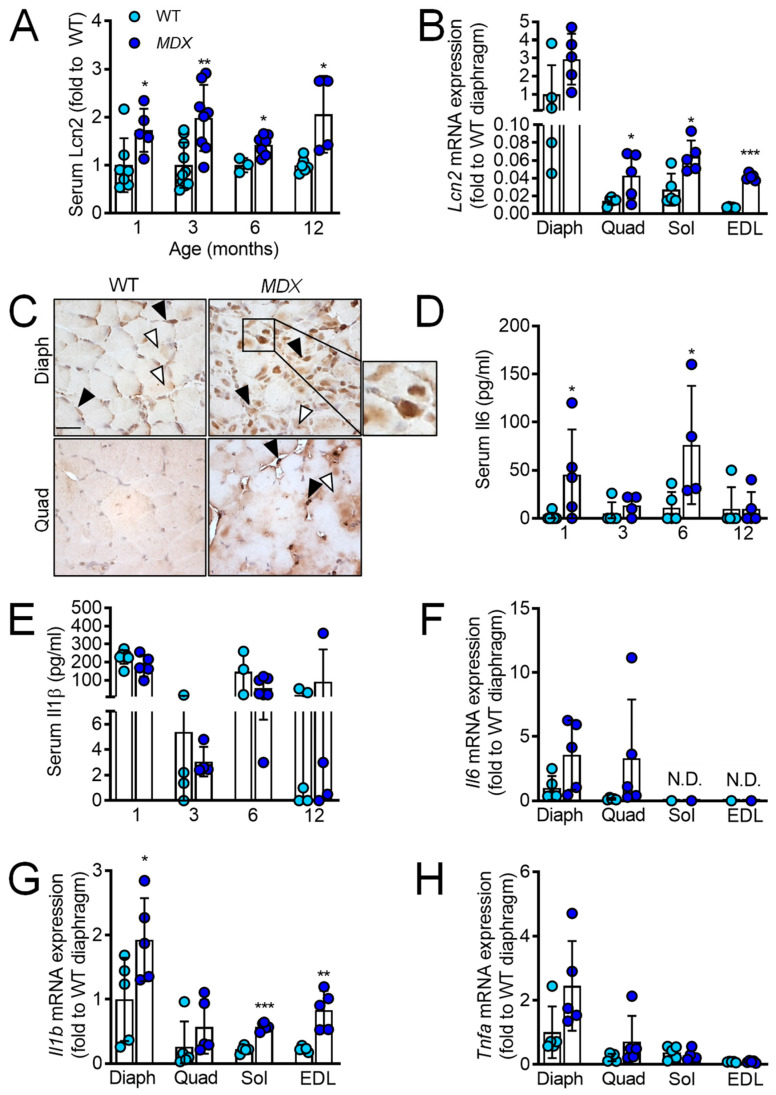
Lipocalin 2 and pro-inflammatory cytokines expression in *MDX* mice. (**A**) ELISA assay showing Lipocalin 2 (Lcn2) serum levels in WT and *MDX* mice at the ages indicated in the abscissa. (**B**) Transcriptional expression of *Lcn2* in *the* diaphragm (Diaph), quadriceps (Quad), *soleus* (Sol), and *Extensor Digitorum Longus* (EDL) muscles from 1-month-old WT and *MDX* mice. (**C**) Immunohistochemical analysis of Lcn2 in diaphragm and quadriceps muscle sections from 1-month-old WT and *MDX* mice (Black arrowhead: mononuclear cells positive for Lcn2; white arrowhead: positivity for Lcn2 in muscle fibres; bar = 25 μm; inset: magnification of Lcn2-positive mononuclear cells). (**D**) ELISA assay to evaluate the serum levels of Interleukin (IL) 6 and (**E**) IL1β at the ages indicated in the abscissa. (**F**) Diaphragm, quadriceps, *soleus* (Sol), and *Extensor Digitorum Longus* (EDL) transcriptional expression of *Il6*, (**G**) *Il1b,* and (**H**) *Tnfa* in 1-month-old WT and *MDX* mice. In (**C**), pictures are representative of three mice/group. In (**A**,**B**,**D**–**H**) results are the mean ± SD of at least three mice/group; N.D. = non-detectable; * *p* < 0.05, ** *p* < 0.01 and *** *p* < 0.001 vs. WT; Student’s *t*-test.

Since Lcn2 is involved in inflammation, to understand its potential role in DMD pathophysiology, we first evaluated its concentration in sera of WT and *MDX* mice, finding that it was significantly higher in the *MDX* mice at 1, 3, 6, and 12 months of age (Figure 1A). We next evaluated Lcn2 transcriptional expression in the diaphragm, quadriceps, *soleus,* and *extensor digitorum longus* (EDL), finding a trend of increase in diaphragm (*p* = 0.07), while it was significantly higher in all other *MDX* muscles compared to WT (Figure 1B). Immunohistochemical analysis confirmed the higher amount of Lcn2 in *MDX* diaphragm and quadriceps, showing that this protein was mainly expressed in mononuclear cells between muscle fibres, although some positivity was also found in the fibres themselves (Figure 1C). Furthermore, while the WT diaphragm showed some positive areas, the WT quadriceps appeared mostly negative (Figure 1C). We then analysed the pro-inflammatory cytokines, finding higher serum levels of IL6 at 1 and 6 months of age in *MDX* mice (Figure 1D) while no differences were observed for IL1β (Figure 1E). At the transcriptional level, *Il6* expression was similar between the two genotypes in quadriceps, undetectable in EDL and *soleus*, while a trend of upregulation was found in *MDX* diaphragm compared to WT (*p* = 0.08, Figure 1F). *Il1b* was significantly higher in the diaphragm, *soleus,* and EDL of *MDX* versus WT, while no differences were observed for quadriceps (Figure 1G). *Tnfa* mRNA did not change between the two genotypes for all the muscles evaluated (Figure 1H). Finally, we found a significant correlation between *Lcn2* and *Il1b* (Supplementary Figure S1A) and *Lcn2* and *Tnfa* (Supplementary Figure S1B) mRNA expression in the diaphragm, quadriceps, EDL, and *soleus* of *MDX* mice, while for WT mice, a positive correlation was only observed between *Lcn2* and *Tnfa* (Supplementary Figure S1C,D). However, the latter correlation has two outliers, and even if statistically significant, it is not clear if it is biologically relevant. Taken together, these results show that Lcn2 is expressed in different muscles, is higher in *MDX* mice compared to WT, and correlates with pro-inflammatory factors in *MDX* mice.

### 2.2. Genetic Ablation of Lcn2 Improves the Bone Phenotype of MDX Mice

To investigate whether Lcn2 could play a role in the osteopenic phenotype observed in DMD, we crossbred *Lcn2^−/−^* mice with *MDX* to obtain *MDX* mice genetically lacking *Lcn2* (*MDXxLcn2^−/−^* mice). Since the three genetically modified mice have different backgrounds (C57BL6 for *Lcn2^−/−^*, C57BL10 for *MDX* and C57BL6 × 10 for *MDXxLcn2^−/−^* mice), data are presented as fold to the appropriate WT, while the raw mean ± SD data are reported in Supplementary Tables SII–SV.

**Figure 2:**
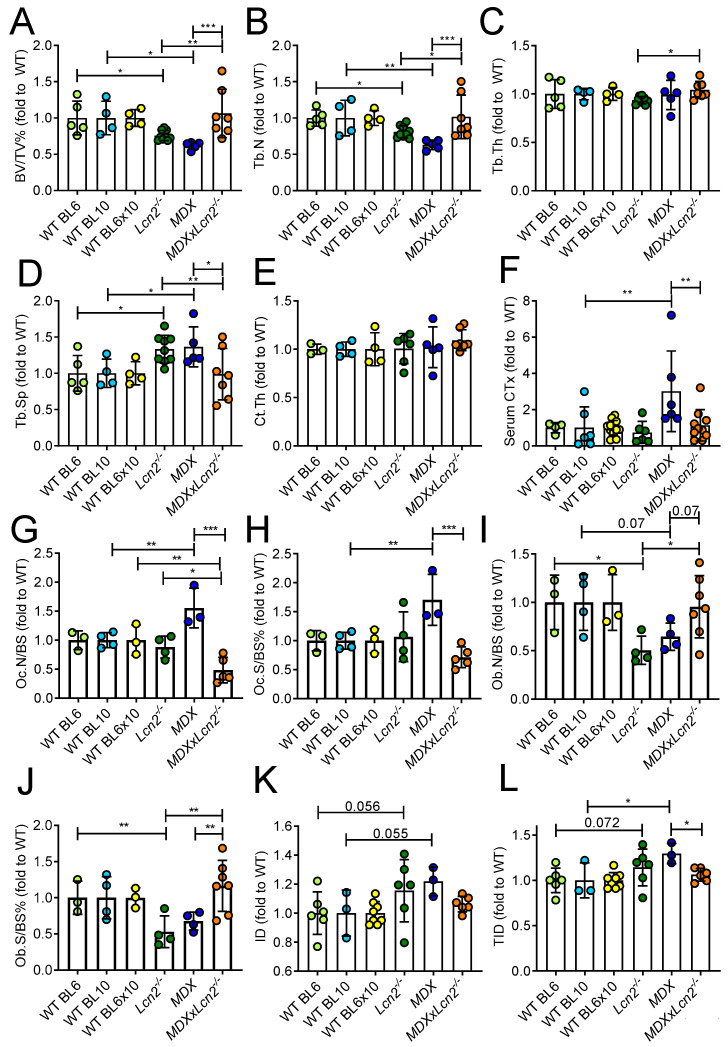
Effect of *Lcn2* genetic ablation on the bone phenotype of 3-month-old *MDX* mice. (**A**) MicroCT analysis performed on proximal tibiae explanted from 3-month-old *Lcn2^−/−^*, *MDX,* and *MDX* mice crossbred with *Lcn2^−/−^* (*MDX*x*Lcn2^−/−^*) and their respective WTs (BL6, BL10, and BL6x10, respectively) to evaluate trabecular bone volume/tissue volume (BV/TV%), (**B**) trabecular number (Tb.N), (**C**) thickness (Tb.Th), and (**D**) separation (Tb.Sp). (**E**) Tibial cortical bone thickness (Ct.Th). (**F**) Serum analysis of carboxy-terminal collagen crosslinks (CTx). (**G**) Histomorphometric analysis of tibia sections to evaluate osteoclast number/bone surface (Oc.N/BS) and (**H**) osteoclast surface/bone surface (Oc.S/BS%) by TRAcP histochemical staining, (**I**) osteoblast number/bone surface (Ob.N/BS), and (**J**) osteoblast surface/bone surface (Ob.S/BS%) by toluidine blue staining. (**K**) Biomechanical test via the Biodent reference point indentation analysis instrument to assess indentation distance (ID) and (**L**) total indentation distance (TID). Results are the mean ± SD of at least three mice/group; * *p* < 0.05, ** *p* < 0.01, and *** *p* < 0.001 between the indicated groups. One-way *ANOVA*.

As expected, micro-computed tomography (μCT) analysis showed that at 3 months of age, *MDX* mice presented with a lower trabecular bone volume/tissue volume (BV/TV) % versus WT BL10, while deletion of Lcn2 in these mice (*MDXxLcn2^−/−^*) increased this parameter (Figure 2A and Supplementary Figure S2) due to an increased trabecular number (Tb.N, Figure 2B), with no difference in trabecular thickness (Tb.Th, Figure 2C). Consistently, trabecular separation (Tb.Sp) was reduced in *MDXxLcn2^−/−^* versus *MDX* (Figure 2D), while cortical thickness (Ct.Th) was not affected (Figure 2E). *Lcn2^−/−^* mice also showed the expected [6] osteopenic phenotype compared to the appropriate WT (BL6) (Figure 2A–D). The rescue of bone mass was at least partially due to a reduction in osteoclast activity, as suggested by the analysis of the bone resorption biomarker CTx, whose serum levels were lower in *MDXxLcn2^−/−^* compared to *MDX* mice (Figure 2F). This was accompanied by a reduction in osteoclast number/bone surface (Figure 2G) and osteoclast surface/bone surface (Figure 2H) in *MDXxLcn2^−/−^* compared to *MDX* mice. Consistent with the CTx, *MDX* mice showed higher osteoclast number and surface, while *MDXxLcn2^−/−^* showed reduced osteoclast number but unremarkable surface versus WT. Nevertheless, we also found involvement of osteoblasts, since toluidine blue staining revealed a trend of increase in osteoblast number/bone surface (Ob.N/BS, Figure 2I) and a significant increase in osteoblast surface/bone surface (Ob.S/BS%, Figure 2J) in *MDXxLcn2^−/−^* mice versus *MDX*. As expected, we also observed a reduction in osteoblast parameters in *Lcn2^−/−^* versus WT BL6, while *MDX* mice showed only a trend of decrease in osteoblast number and no significant difference in osteoblast surface versus WT BL10.

Next, we analysed the bone mechanical properties by reference point indentation (RPI) analysis assessing the indentation distance (ID) and the total indentation distance (TID) in tibiae, which are inversely correlated with the mechanical strength of the bone. ID was not significantly affected in any of the groups, although a trend of increase was found in *Lcn2^−/−^* and *MDX* versus their WT (*p* = 0.056 and *p* = 0.055, respectively, Figure 2K), while TID was significantly increased in *MDX*, hence demonstrating worse mechanical properties (Figure 2L). This impairment was significantly blunted in *MDXxLcn2^−/−^,* thus restoring the mechanical properties near to that of WT mice (Figure 2L). Non-normalised data of all groups presented in Figure 2 are shown in Supplementary Table SII.

**Figure 3:**
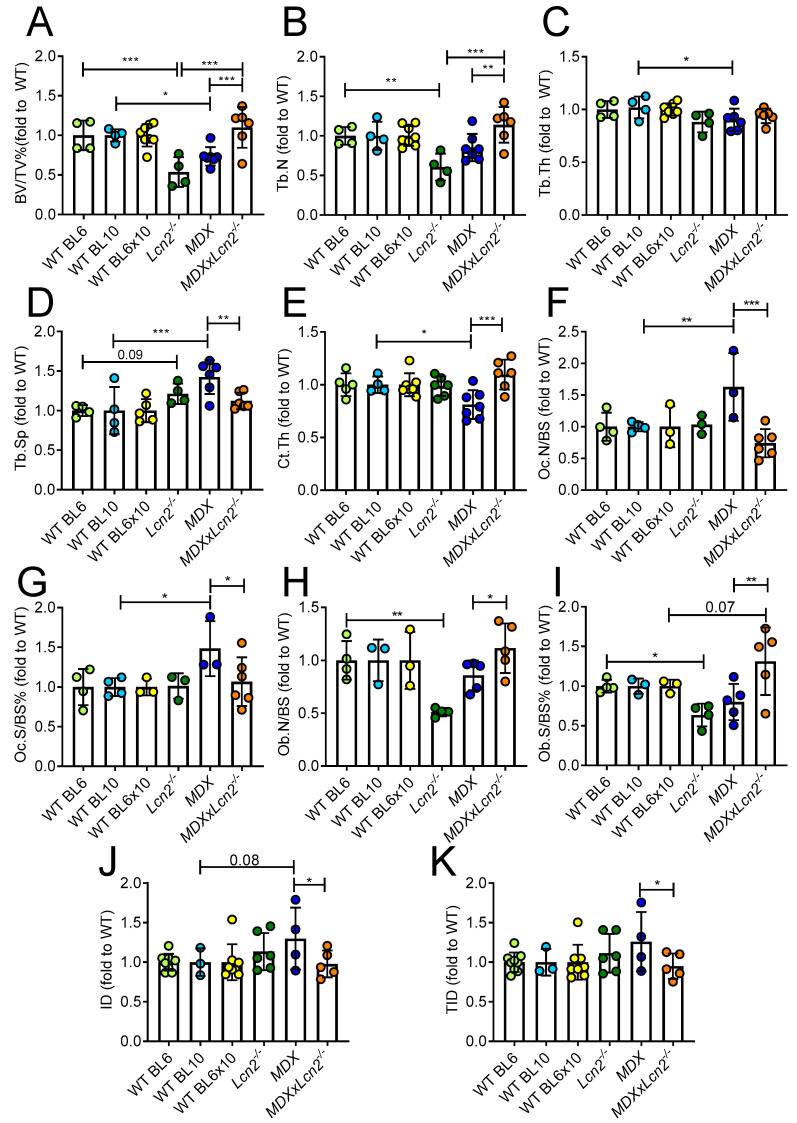
Effect of *Lcn2* genetic ablation on the bone phenotype of 6-month-old *MDX* mice. (**A**) MicroCT analysis performed on proximal tibiae explanted from 6-month-old *Lcn2^−/−^*, *MDX,* and *MDX* mice crossbred with *Lcn2^−/−^* (*MDX*x*Lcn2^−/−^*) and their respective WTs (BL6, BL10, and BL6x10, respectively) to evaluate trabecular bone volume/tissue volume (BV/TV%), (**B**) trabecular number (Tb.N), (**C**) thickness (Tb.Th), and (**D**) separation (Tb.Sp). (**E**) Tibial cortical bone thickness (Ct.Th). (**F**) Histomorphometric analysis of tibia sections to evaluate osteoclast number/bone surface (Oc.N/BS) and (**G**) osteoclast surface/bone surface (Oc.S/BS%) by TRAcP activity histochemical staining, (**H**) osteoblast number/bone surface (Ob.N/BS), and (**I**) osteoblast surface/bone surface (Ob.S/BS%) by toluidine blue staining. (**J**) Tibiae biomechanical test by Biodent reference point indentation analysis instrument, to assess (**J**) indentation distance (ID) and (**K**) total indentation distance (TID). Results are the mean ± SD of at least three mice/group; * *p* < 0.05, ** *p* < 0.01 and *** *p* < 0.001 between the indicated groups. One-way *ANOVA*.

At 6 months of age, we also found *MDX* mice to be osteopenic and, similarly to what was observed at 3 months of age, a rescue of the bone phenotype was observed when genetically ablating *Lcn2* from *MDX* mice. In particular, bone volume was increased and returned to WT levels (Figure 3A and Supplementary Figure S2), paralleled by an increase in trabecular number (Figure 3B), with no significant differences in trabecular thickness (Figure 3C) and reduced trabecular separation (Figure 3D) in *MDXxLcn2^−/−^* mice versus *MDX*. Moreover, *MDXxLcn2^−/−^* showed significantly higher Ct.Th compared to *MDX* alone (Figure 3E). As expected, osteoclast variables were increased in *MDX* mice, as analysed by histomorphometry following TRAcP activity histochemical detection (Supplementary Figure S3A), which showed a significantly higher osteoclast number/bone surface (Oc.N/BS, Figure 3F) and osteoclast surface/bone surface (Oc.S/BS %, Figure 3G) in *MDX* mice versus WT. Interestingly, *MDXxLcn2^−/−^* mice showed a significant reduction in these variables versus *MDX*. Moreover, osteoblast variables, evaluated by toluidine blue staining (Supplementary Figure S3B), were affected, with a significantly higher osteoblast number/bone surface (Ob.N/BS, Figure 3H) and osteoblast surface/bone surface (Ob.S/BS%, Figure 3I) in *MDXxLcn2^−/−^* mice versus *MDX*. As expected, we also observed a reduction in osteoblast parameters in *Lcn2^−/−^* versus WT BL6. As for the evaluation of mechanical properties, ID showed a trend of increase in *MDX* mice versus WT (*p* = 0.08), which is consistent with what was observed at 3 months of age. The deletion of *Lcn2* resulted in a significant reduction of ID and TID in *MDXxLcn2^−/−^* mice compared to *MDX* (Figure 3J,K), thus confirming the improvement of the bone mechanical properties induced by *Lcn2* genetic ablation. Non-normalised data of all groups presented in Figure 3 are shown in Supplementary Table SIII. Of note, most of the genotype-associated changes found in younger mice were not present at 12 months of age (Supplementary Figures S2 and S4). Indeed, none of the μCT variables analysed were significantly different between *MDXxLcn2^−/−^* and *MDX* mice (Supplementary Figure S4), but it is worth noting that at this age, *MDX* mouse bone variables were unremarkable compared to WT as well. Interestingly, BV/TV and Tb.N were significantly higher, and Tb.Sp significantly lower in *MDXxLcn2^−/−^* mice versus *Lcn2^−/−^* (Supplementary Figure S4). Non-normalised data of all groups are shown in Supplementary Table SIV.

### 2.3. Genetic Ablation of Lcn2 Improves the Muscle Phenotype of MDX Mice

**Figure 4:**
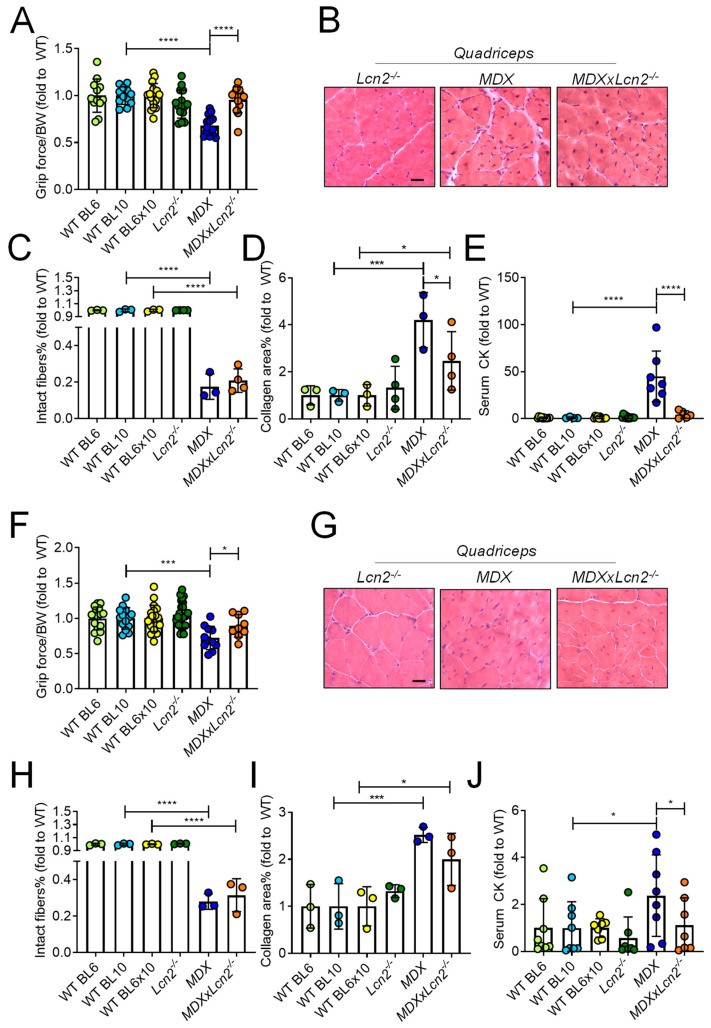
Effect of *Lcn2* genetic ablation on the muscle phenotype of *MDX* mice. (**A**) Three-month-old *Lcn2^−/−^*, *MDX,* and *MDX* mice crossbred with *Lcn2^−/−^* (*MDX*x*Lcn2^−/−^*) and their respective WTs (BL6, BL10, and BL6x10, respectively) were subjected to grip force test to evaluate muscle performance. (**B**) Haematoxylin-eosin staining of muscle sections to assess (**C**) the % of intact fibres. Bar = 30 μm. (**D**) Percent of collagen area quantified in Masson’s trichrome-stained muscle sections. (**E**) ELISA assay to quantify serum creatine kinase (CK). (**F**) Six-month-old *Lcn2^−/−^, MDX,* and *MDX*x*Lcn2^−/−^* mice were subjected to grip force test, and (**G**) haematoxylin/eosin staining of muscle sections was performed to assess (**H**) % of intact fibres. (**I**) Percent of collagen area quantified in Masson’s trichrome-stained muscle sections. (**J**) ELISA assay to quantify serum creatine kinase (CK). Results are the mean ± SD of at least three mice/group; * *p* < 0.05, *** *p* < 0.001, and **** *p* < 0.0001 between the indicated groups. One-way *ANOVA*.

We next evaluated the effect of Lcn2 ablation on the *MDX* muscle phenotype. The grip force test performed at 3 months of age showed an impairment of muscle performance in *MDX* mice compared to WT, which was completely abrogated by *Lcn2* deletion (Figure 4A, Supplementary Table SV). We then analysed the muscle phenotype histologically. Haematoxylin-eosin staining (Figure 4B) showed that the percentage of intact fibres with no central nuclei was significantly lower in both *MDX* and *MDXxLcn2^−/−^* mice compared to WT and *Lcn2^−/−^* mice (Figure 4C and Supplementary Table SV), thus indicating that *Lcn2* ablation did not improve this parameter in *MDX* mice. We noticed a reduced collagen area, evaluated by Masson’s trichrome staining in the quadriceps of *MDXxLcn2^−/−^* mice versus *MDX*, which was, however, still significantly higher compared to WT (Figure 4D and Supplementary Table SV). Despite this, a significant reduction of the muscle damage marker, creatine kinase (CK), was observed in *MDXxLcn2^−/−^* mice compared to *MDX*, the latter also being significantly higher compared to the respective WT (Figure 4E and Supplementary Table SV). We then analysed 6-month-old mice, finding again an impairment of muscle function, evaluated by a grip force test, in *MDX* mice compared to WT, which was reversed in *MDXxLcn2^−/−^* (Figure 4F and Supplementary Table SV). Histological analysis, performed on haematoxylin-eosin stained muscle sections (Figure 4G), showed a reduction of the percentage of intact fibres in both *MDX* and *MDXxLcn2^−/−^* mice quadriceps versus WT, while *Lcn2^−/−^* mice had a significantly higher percentage of intact fibres compared to *MDXxLcn2^−/−^* mice (Figure 4H). Quadriceps fibrosis was also higher in both *MDX* and *MDXxLcn2^−/−^* compared to their respective WT (Figure 4I), suggesting that the partial rescue was observed at 3 months of age in *MDXxLcn2^−/−^*, whereas *MDX* faded with ageing. Serum analysis showed significantly higher CK levels in *MDX* compared to WT (Figure 4J and Supplementary Table SV) and *MDXxLcn2^−/−^* mice (Figure 4J).

### 2.4. Lcn2 Blockade via a Monoclonal Antibody Improves Bone and Muscle Phenotype of MDX Mice

**Figure 5:**
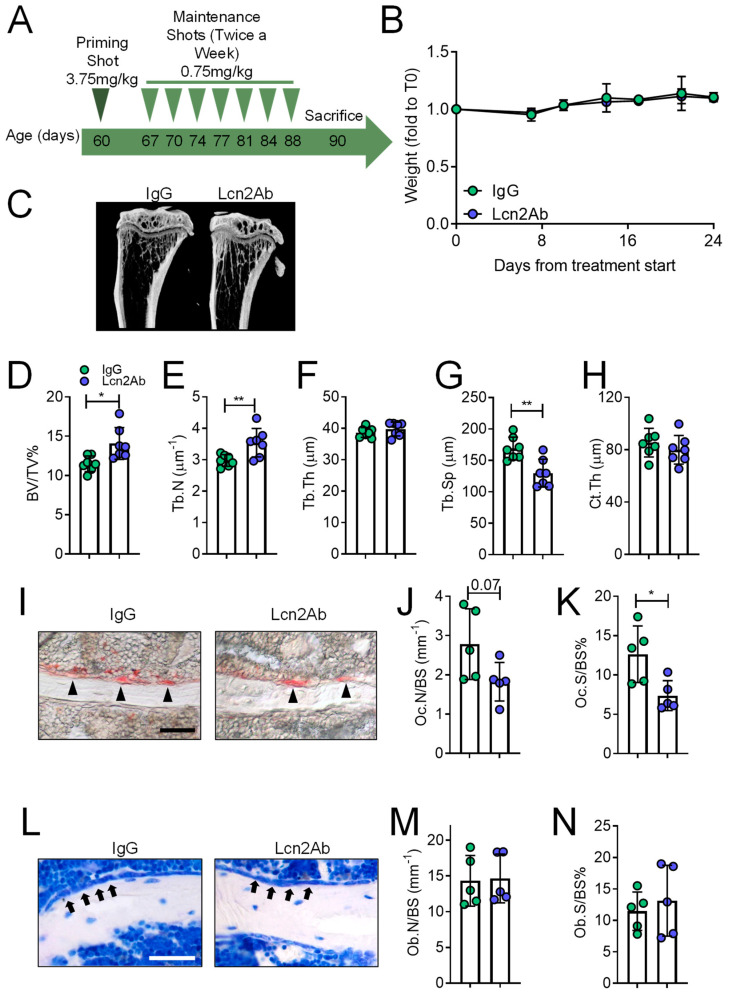
Treatment of *MDX* mice with anti-Lcn2 antibody: bone phenotype. (**A**) Schematic representation of the treatment of 2-month-old *MDX* male mice with an anti-Lcn2 antibody (Lcn2Ab) or with an irrelevant IgG (IgG), according to a curative protocol. (**B**) Evaluation of body weight during the timeframe of the experiment. (**C**) MicroCT analysis of the proximal tibiae to evaluate trabecular (**D**) bone volume/tissue volume (BV/TV%), (**E**) number (Tb.N), (**F**) thickness (Tb.Th), (**G**) separation (Tb.Sp), and (**H**) tibial cortical thickness (Ct.th). (**I**) Histochemical assay of TRAcP activity in tibia sections to assess (**J**) osteoclast number/bone surface (Oc.N/BS) and (**K**) osteoclast surface/bone surface (Oc.S/BS%). (**L**) Toluidine blue staining of tibia sections to evaluate (**M**) osteoblast number/bone surface (Ob.n/BS) and (**N**) osteoblast surface/bone surface (Ob.S/BS%). Results are the mean ± SD of at least five mice/group; Bar = 30 μm. * *p* < 0.05; ** *p* < 0.01; Student’s *t*-test.

To validate the results obtained with mouse genetics, we treated *MDX* mice with a commercially available Lcn2-blocking monoclonal antibody (Lcn2Ab) using a curative treatment. Briefly, we administered, by intraperitoneal injection, a priming shot of 3.75 mg/Kg to 2-month-old *MDX* mice, which already manifested the DMD-like phenotype, followed by maintenance shots of 0.75 mg/Kg administered twice a week until sacrifice at 3 months of age (Figure 5A). Control mice were treated identically but with an irrelevant IgG. We also measured the body weight during the timeframe of the experiment, which was unremarkable between the two treatments (Figure 5B). µCT analysis of the proximal tibia trabecular bone (Figure 5C) showed that the treatment with Lcn2Ab increased BV/TV % (Figure 5D) and Tb.N (Figure 5E), while Tb.Th was unaffected (Figure 5F); Tb.Sp was decreased (Figure 5G) compared to treatment with irrelevant IgG. This was consistent with what we observed using mouse genetics. Ct.Th was instead unremarkable between the two groups (Figure 5H). Moreover, histomorphometry showed a decreasing trend (*p* = 0.07) of the osteoclast number in Lcn2Ab-treated mice (Figure 5I,J), while osteoclast surface was significantly reduced (Figure 5K). Toluidine blue staining (Figure 5L) showed that the osteoblast number (Figure 5M) and surface (Figure 5N) were similar in control and treated mice.

**Figure 6:**
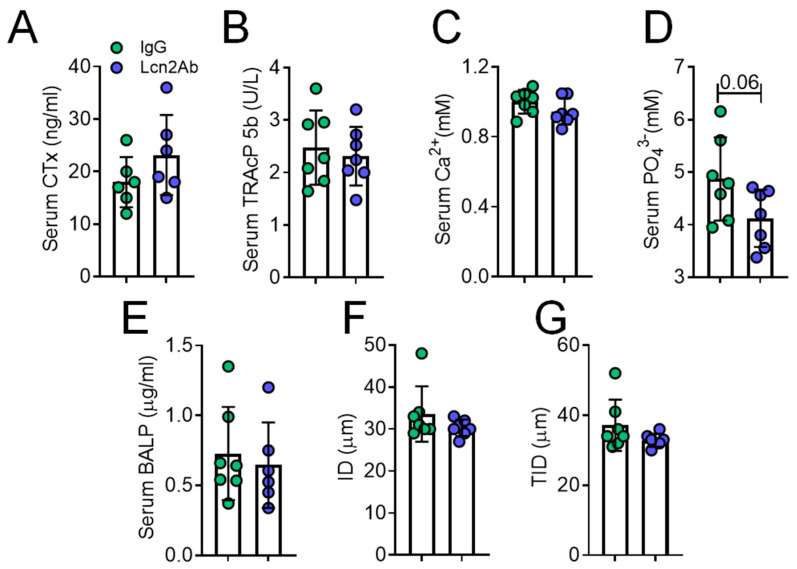
Treatment of *MDX* with anti-Lcn2 antibody: serum variables. Two-month-old *MDX* mice were treated with an anti Lcn2 antibody (Lcn2Ab) or an irrelevant IgG (IgG), as shown in Figure 5A. At the end of the experiment, sera were harvested to evaluate (**A**) carboxy-terminal collagen crosslinks (CTx), (**B**) tartrate-resistant acid phosphatase (TRAcP) 5b, (**C**) calcium and (**D**) phosphate ions, and (**E**) bone-specific alkaline phosphatase (BALP). (**F**) Tibiae were explanted and subjected to biomechanical testing via the Biodent reference point indentation analysis instrument to assess indentation distance and (**G**) total indentation distance. Results are the mean ± SD of at least six mice/group; Student’s *t*-test.

Serum bone turnover markers, CTx (Figure 6A), TRAcP 5b (Figure 6B), and free Ca^2+^ (Figure 6C) were unremarkable. Serum phosphate showed a trend of decrease following the treatment with Lcn2Ab (*p* = 0.07, Figure 6D), while the serum osteoblast marker bone alkaline phosphatase (BALP) was not affected by the treatment (Figure 6E). Moreover, treatment with anti Lcn2Ab did not improve the bone biomechanical parameters’ ID (Figure 6F) and TID (Figure 6G).

**Figure 7:**
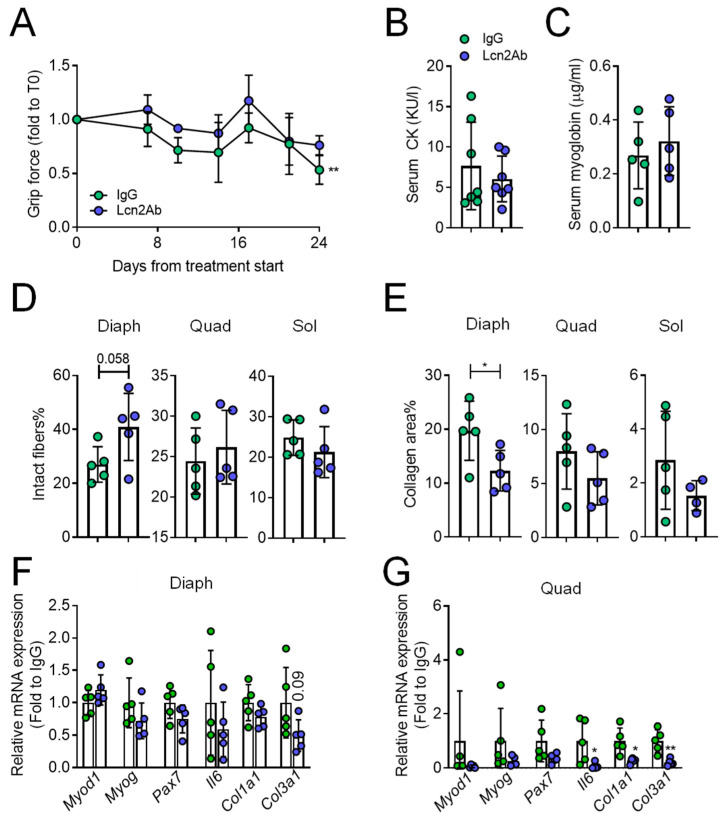
Treatment of *MDX* mice with anti-Lcn2 antibody: muscle phenotype. (**A**) Evaluation of muscle performance of *MDX* mice treated with irrelevant IgG (IgG) or with an anti-Lcn2 antibody (Lcn2Ab) during the timeframe of the experiment. (**B**) Reflotron and ELISA assays to evaluate serum levels of creatine kinase (CK) and (**C**) myoglobin, respectively. (**D**) Explanted diaphragm (Diaph), quadriceps (Quad), and *soleus* (Sol) were snap-frozen without fixation and embedded in OCT. After sectioning, muscles were subjected to haematoxylin/eosin staining to assess % of intact fibres and (**E**) to Masson’s trichrome staining to evaluate fibrosis. (**F**) Diaphragm and (**G**) quadriceps were explanted and subjected to RNA extraction, cDNA synthesis and comparative transcriptional analysis via real-time PCR. The genes analysed were *myogenic differentiation d1* (*Myod1*), *myogenin* (*Myog*), *paired box protein 7* (*Pax7*), *interleukin* (*Il)6*, *collagen* (*Col)1a1,* and *Col3a1*. Results are the mean ± SD of at least four mice/group. In (**A**), curve fitting test. In (**B**–**G**) Student’s *t*-test. * *p* < 0.05; ** *p* < 0.01 vs. IgG.

With regard to the muscle phenotype, grip force, evaluated during the timeframe of the experiment, showed a significant increase in Lcn2Ab-treated mice (Figure 7A), consistent with what we found in the double mutant studies. However, no differences were observed in CK (Figure 7B) and myoglobin (Figure 7C) serum levels. Haematoxylin/eosin staining showed that the diaphragm had a trend of increase in intact fibres (*p* = 0.06), while the other muscles were unaffected in Lcn2Ab- versus IgG-treated *MDX* mice (Figure 7D). Fibrosis, evaluated by Masson’s trichrome staining, was significantly lower in the diaphragm of Lcn2Ab-treated *MDX* mice versus IgG, while no differences were found in the quadriceps and *soleus* (Figure 7E). Finally, we performed a transcriptional analysis in the diaphragm, finding no differences of the myogenic factors *Myogenic differentiation d1* (*Myod1*) and *Myogenin* (*Myog*), and the satellite cell marker *Paired box protein 7* (*Pax7*) and *Il6* (Figure 7F). However, while *Col1a1* showed no difference between IgG and Lcn2Ab treatment, *Col3a1*, the main fibrillar collagen in muscle, showed a trend of decrease (*p* = 0.09) following Lcn2Ab administration (Figure 7F). Similar transcriptional analysis in the quadriceps revealed no differences in *Myod1*, *Myog*, and *Pax7*, but a significant reduction of *Il6*, *Col1a1,* and *Col3a1* in Lcn2Ab-treated *MDX* mice compared to IgG (Figure 7G).

In addition to its efficacy, it is important to test a treatment’s safety for the other organs. Hence, we ran key analyses to analyse liver and kidney damage in MDX mice treated with IgG or Lcn2Ab. Treatment with Lcn2Ab did not affect the kidney, as demonstrated by microscopical examination of hematoxylin-eosin stained sections (Supplementary Figure S5A) and histopathological assessment of the kidney collagen area (Supplementary Figure S5B). Consistently, the serum marker uric acid was similar between the two groups (Supplementary Figure S5C). Liver health was also unaffected by the treatment (Supplementary Figure S5D). Evaluation of the collagen area (Supplementary Figure S5E) and serum analysis of Alanine Transaminase (ALT, Supplementary Figure S5F) showed a trend of increase in the former and a significant increase in the latter in *MDX* mice, which is consistent with previous findings [25,26]. However, treatment with Lcn2Ab did not seem to worsen liver damage.

Taken together, our results demonstrate that the genetic deletion of Lcn2 in *MDX* mice improved their bone health and had beneficial effects on muscle performance until 6 months of age, but not further on. These effects seemed to be partially confirmed by blocking Lcn2 with a monoclonal antibody.

## 3. Discussion

In this paper, we show that the genetic ablation of Lcn2 or the inhibition of its function by an Lcn2-blocking antibody in the *MDX* mouse model of DMD can counteract bone loss. This is in agreement with the fact that higher Lcn2 levels are observed in femurs and bone cells in conditions of mechanical unloading and simulated microgravity, respectively [4,27], and strengthen the concept that in conditions of mechanical unloading/disuse such as DMD, Lcn2 is a detrimental factor for bone mass. It is very hard to determine specifically whether inflammation, disuse, or muscle damage are causing elevated Lcn2 serum levels, but given the literature data and our own observations, one could speculate that all of these factors coexisting in *MDX* mice may contribute to increasing Lcn2 serum levels. Of note, *Lcn2^−/−^* mice are osteopenic [6]. This apparent discrepancy could be explained by acknowledging that Lcn2 may have two distinct roles in physiological versus inflammatory conditions. In *MDX* mice, characterised by muscle damage and flogosis, the “pro-inflammatory” Lcn2 might be more important, and the osteopenia-inducing effect of the lack of Lcn2 is irrelevant compared to the damage it causes. The situation is complicated by the fact that *Lcn2^−/−^* mice also have other minor phenotypes, such as altered energy metabolism and glucose transporters gene expression [6], reduced muscle fibre size, and when subjected to mechanical unloading, they lose less mass in some of their muscles, and more in others compared to WT [9]. Although complex, the net result of the regulatory mechanisms driven by the lack of Lcn2 in *MDX* mice is a phenotypical improvement in bone, and at least partially, in muscle.

In muscle, the general improvement of the phenotype in *MDX* mice by Lcn2 ablation is not completely unexpected since the reduced inflammation correlated with Lcn2 [4,28,29,30] may lead to less fibrosis and reduced muscle damage [31]. Moreover, in a recent report, we showed that *Lcn2^−/−^* mice subjected to mechanical unloading do not lose muscle function, at least in the short-medium term [9], which is consistent with the grip force improvement we observed in the *MDXxLcn2^−/−^* mice. Furthermore, treating myoblasts with recombinant Lcn2 in vitro reduced their myogenic differentiation [9]. The fact that at 12 months of age, the phenotypical improvements provided by Lcn2 removal are no longer observed is consistent with the possibility that the damage is not entirely prevented but only delayed.

Interestingly, some advantages of Lcn2 ablation in *MDX* mice can also be reproduced by treating *MDX* mice with a blocking Lcn2Ab. It is worth noting that while removing Lcn2 genetically could be considered a preventive regulation, our antibody treatment protocol was curative, as it was started at 2 months of age when the dystrophic disease peak already took place and caused damage. Another possible advantage of using Lcn2Ab in DMD is that recent reports suggest a link between glucocorticoids and Lcn2. In particular, Conde and colleagues [32] suggested that glucocorticoids induce Lcn2 expression through IL1. This would be consistent with our data showing that *Il1b* correlates with *Lcn2*, at least in muscle. If this observation is confirmed in DMD, it would strengthen the rationale for an anti-Lcn2 treatment to limit the side effects of glucocorticoids treatment on bone.

In conclusion, we showed that deleting Lcn2, genetically or blocking its activity with a monoclonal antibody, in the *MDX* mouse model of DMD is able to prevent or revert the bone loss induced by this disease. Muscle health is also improved when removing Lcn2, although the phenotype is still far from being considered normal from a muscle standpoint. Therefore, Lcn2 removal is worthy of further investigation as part of a therapy program to treat DMD-induced bone loss while also helping to improve muscle health.

## 4. Materials and Methods

### 4.1. Materials

TRIzol reagent, primers, and reagents for RT-PCR were from Invitrogen (Carlsbad, CA, USA). The Sensimix SYBR Green QPCR master mix was from Bioline (Memphis, TN, USA). Masson’s trichrome kit and the other histological supplies were from Bio Optica (Milan, Italy). ELISA kits for Lcn2 were from R&D (cat# MLCN20, Minneapolis, MN, USA), for myoglobin (cat# ab210965) and interleukin (IL)6 (cat# ab100712) from Abcam (Cambridge, UK), for IL1β (cat# ELM-IL1b-1) from Raybiotech (Atlanta, GE, USA), for bone alkaline phosphatase (BALP, cat# CSB-E11914m) from Cusabio (Houston, TX, USA), and for carboxy-terminal collagen crosslinks (CTx) EIA (Cat#AC-06F1, RRID:AB_2801265) from IDS (The Boldons, UK). Reflotron PLUS reactive strips to evaluate creatine kinase (CK) were from Roche (Basel, Switzerland). Reagents to quantify calcium concentration were from Randox (Crumlin, UK), while the phosphate evaluation kit (cat# ab65622) was from Abcam. The antibodies to neutralise Lcn2 in *MDX* mice and for immunohistochemical detection of Lcn2 were from R&D (cat# MAB18571, RRID:AB_10890764 and AF1857, RRID:AB_355022, respectively), while irrelevant IgG2A was from Santa Cruz Biotechnology (cat# sc-3883, RRID:AB_737252). All other reagents were of the purest grade from Sigma Aldrich Co. (St. Louis, MO, USA).

### 4.2. Study Approval

Procedures involving animals were approved by the Italian Ministry of Health (ethical approval protocol N.365/2017-PR).

### 4.3. Animals

*Lcn2^−/−^* mice (background C57BL6/J, IMSR Cat# JAX:000664, RRID:IMSR_JAX:000664) were bred and kindly provided by Dr. Tak Wah Mak (University Health Network, Toronto, ON, Canada) [33]. *MDX* (IMSR Cat# JAX:001801, RRID:IMSR_JAX:001801) and C57BL10 (IMSR Cat# JAX:000476, RRID:IMSR_JAX:000476) mice were purchased from the Jackson laboratory, Bar Harbor, ME, USA. *MDXxLcn2^−/−^* and C57BL6x10 mice, the latter generated to match the genetic background of the former, were obtained by in-house crossbreeding. All procedures involving animals and their care were conducted in conformity with national and international laws and policies (European Economic Community Council Directive 86/609, OJ L 358, 1, 12 December 1987; Italian Legislative Decree 4.03.2014, n.26, *Gazzetta Ufficiale della Repubblica Italiana* no. 61, 4 March 2014) and the Animal Research: Reporting of In Vivo Experiments (ARRIVE) guidelines. Mice were housed in the animal facility of the University of L’Aquila, Italy, at the following conditions: temperature: 20–24 °C, humidity: 60%, dark/light cycle: 12/12 h. They had access to food and water ad libitum and were fed with a standard diet (Mucedola, Milan, Italy. Code: 4RF21) composed of 60.8% carbohydrates, 21% proteins, 3.45% fat, 6.8% fibres, 7.95% trace elements, and 12% humidity.

### 4.4. Forelimb Grip Strenght Tests

Mice were acclimated in the experimental room overnight, and tests were run in the morning using a grip strength meter apparatus with a mouse forelimb grip attachment (Ugo Basile, Varese, Italy). Tests were performed according to the TreatNMD DMD_M.2.2.001. Briefly, mice were lifted by the tail and gradually drew closer to the grip bar from above until they grasped it firmly. Then they were slowly pulled back with constant force until they released the grasp. Four-5 determinations per mouse were performed, at least 1 min apart from each other and the mean grip force (measured in grams-force) among all determinations was calculated. Mice were then weighed and returned to their cages. Finally, grip force normalised by body weight was calculated.

### 4.5. Anti-Lcn2 Antibody Treatment

The antibody used to neutralise Lcn2 in vivo was commercially available and has previously been used to this end by other investigators. According to Cheng et al. [34] and Pelosi et al. [35], we used a priming dose of 3.75 mg/kg and seven maintenance doses of 0.75 mg/kg each, starting 1 week after the priming shot, administered twice a week. Dilutions were prepared in 0.9%NaCl just before the shot, after weighing the mice. The injection route for all treatments was intraperitoneal. The treatment was administered to 2-month-old mice and lasted 30 days in total.

### 4.6. Comparative Real-Time RT-PCR

Total RNA was extracted from mouse muscles using the TRIzol^®^ method. One microgram of RNA was reverse transcribed into cDNA using Moloney Murine Leukemia Virus (M-MLV) reverse transcriptase, and the equivalent of 0.1 μg was processed using the Sensimix SYBR Green QPCR master mix for real-time PCR. Results, expressed as fold-changes versus WT average using the ΔΔCt method, were normalised with the housekeeping gene glycerol-3-phosphate dehydrogenase (*Gapdh*). The list of primers used in the study is available in Supplementary Table SI.

### 4.7. Histology and Histopathology

Diaphragm (Diaph), *Quadriceps* (Quad), *Soleus* (Sol), and *Extensor Digitorum Longus* (EDL) muscles were isolated from euthanised mice, equalised in OCT medium for 10 min, put into cryomolds, and snap-frozen in liquid nitrogen-cooled isopentane before storage at −80 °C. Seven μm-thick sections were obtained using a Leica CM1850 cryostat and employed immediately for staining with haematoxylin-eosin or Masson’s trichrome kit (Bio Optica). Sections were then dehydrated and mounted with permanent medium (Eukitt). To quantify the intact fibres, we enumerated those with no central nuclei and reported them as a % of the total fibres in haematoxylin-eosin stained sections. To calculate the % of collagen or fibrotic tissue, we used ImageJ software to select fibrotic tissue in muscles following Masson’s trichrome staining and calculated the % of fibrotic area over the total area per section [36].

Histopathological analysis of the liver and kidney collected from IgG- or Lcn2 antibody-treated mice was carried out in 4% paraformaldehyde (PFA) fixed and paraffin-embedded samples sectioned at 5 μm and stained with haematoxylin-eosin or Masson’s trichrome as described.

Immunohistochemistry for Lcn2 was performed using 5 μm-thick sections of 4% PFA fixed and paraffin-embedded quadricep or diaphragm. Antigen retrieval was performed using pH 6.0 sodium citrate and the detection kit from Vector Laboratories (cat# PK-6105).

### 4.8. Bone Histomorphometry

Tibiae explanted from euthanised mice were fixed in 4% PFA, dehydrated in ascending alcohol series, and processed for methyl–methacrylate embedding without decalcification. Histomorphometric measurements were carried out on 5 μm-thick sections using NIH ImageJ (RRID:SCR_003070) version 1.50i and with the suggested nomenclature [37]. Osteoclast number/bone surface (Oc.N/BS) and osteoclast surface/bone surface (Oc.S/BS%) were evaluated after histochemical staining for tartrate-resistant acid phosphatase (TRAcP) activity. Osteoblast number/bone surface (Ob.N/BS) and osteoblast surface/bone surface (Ob.S/BS%) were evaluated in sections stained with toluidine blue.

### 4.9. Micro Computed Tomography (µCT) Analysis

Images of tibiae previously fixed in 4% PFA were acquired using the SkyScan 1174 (Bruker, Billerica, MA, USA) with a resolution of 6.7 μm (X-ray voltage 50 kV). Image reconstruction was carried out employing a modified Feldkamp algorithm [38] and Skyscan Nrecon software. Three-dimensional (3D) and two-dimensional (2D) morphometric parameters were calculated for the trabecular bone, 100 slides (6.7 μm thick) from the growth plate [23]. Three-dimensional parameters were based on the analysis of a Marching Cubes-type model with a rendered surface [39]. The calculation of all 2D areas and perimeters was based on the Pratt algorithm [40]. Bone structural variables and nomenclature were those suggested by Bouxsein and colleagues [41]. Cortical bone thickness was analysed 450 slides below the growth plate on 54 slides as described [23].

### 4.10. Biodent^®^ Mechanical Testing

Tibiae were harvested from treated mice, cleaned free of soft tissues, and stored at −80 °C. After thawing, the mechanical test was performed on the distal portion of the tibia, immediately above the insertion of the fibula, using the Reference Point Indentation (RPI) technique by Biodent^®^ (Active Life Scientific, Santa Barbara, CA, USA). Bones were kept in ice-cold PBS during the test to maintain tissue hydration. All the samples were tested using 5 to 10 indentation cycles at 2 Hertz (Hz) utilising a force of 2 or 4 Newton (N) depending on the age of the mice. Indentation distance (ID) and total indentation distance (TID) were calculated for each test following the software’s instructions.

### 4.11. Statistics

Results were expressed as the mean ± SD. Correlation analyses were performed using Pearson’s correlation test (R and *p* values are indicated in the graphs). To compare curves in longitudinal studies, Graphpad Prism (RRID:SCR_002798, version 7.0) was used to run curve fitting tests and evaluate whether one curve could fit the datasets compared. A Shapiro–Wilk normality test was performed to assess whether a parametric or non-parametric test was more appropriate in all analyses performed. In experiments with more than two independent experimental groups, one-way ANOVA (parametric) or Kruskal–Wallis (non-parametric) was used to calculate significance. An unpaired Student’s *t*-test (parametric) or Mann–Whitney (non-parametric) test was used when comparing two groups. The statistic tests used are specified in figure legends. For the analysis of the data presented in Figure 2, Figure 3, Figure 4 and Figure 5, we first normalised all raw values for the average of the appropriate WT, pooled all data from WTs, genetically modified mice in the same dataset, and analysed them using multi-group analysis as described above. For the sake of clarity, we then only reported the normalised values in the main figures, while the raw values of all groups are presented in the Supplementary tables. The statistical unit of the study is the mouse, and every dot in all graphs except the line graphs represent a mouse. In all other instances, the number of mice is specified in the figure legends. A *p*-value < 0.05 was considered statistically significant.

## Data Availability

Data is available with the corresponding author upon reasonable request.

## Supplementary Materials

The following are available online at https://www.mdpi.com/article/10.3390/ijms23020958/s1.

## References

[1] Lögdberg L., Wester L. (2000). Immunocalins: A lipocalin subfamily that modulates immune and inflammatory responses. Biochim. Biophys. Acta Protein Struct. Mol. Enzymol..

[2] Mishra J., Qing M.A., Prada A., Mitsnefes M., Zahedi K., Yang J., Barasch J., Devarajan P. (2003). Identification of neutrophil gelatinase-associated lipocalin as a novel early urinary biomarker for ischemic renal injury. J. Am. Soc. Nephrol..

[3] Costa D., Lazzarini E., Canciani B., Giuliani A., Spanò R., Marozzi K., Manescu A., Cancedda R., Tavella S. (2013). Altered bone development and turnover in transgenic mice over-expressing Lipocalin-2 in bone. J. Cell. Physiol..

[4] Rucci N., Capulli M., Piperni S.G., Cappariello A., Lau P., Frings-Meuthen P., Heer M., Teti A. (2015). Lipocalin 2: A new mechanoresponding gene regulating bone homeostasis. J. Bone Miner. Res..

[5] Mosialou I., Shikhel S., Liu J.M., Maurizi A., Luo N., He Z., Huang Y., Zong H., Friedman R.A., Barasch J. (2017). MC4R-dependent suppression of appetite by bone-derived lipocalin 2. Nature.

[6] Capulli M., Ponzetti M., Maurizi A., Gemini-Piperni S., Berger T., Mak T.W., Teti A., Rucci N. (2018). A Complex Role for Lipocalin 2 in Bone Metabolism: Global Ablation in Mice Induces Osteopenia Caused by an Altered Energy Metabolism. J. Bone Miner. Res..

[7] Gambara G., Salanova M., Ciciliot S., Furlan S., Gutsmann M., Schiffl G., Ungethuem U., Volpe P., Gunga H.C., Blottner D. (2017). Microgravity-induced transcriptome adaptation in mouse paraspinal longissimus dorsi muscle highlights insulin resistance-linked genes. Front. Physiol..

[8] Rebalka I.A., Monaco C.M.F., Varah N.E., Berger T., D’souza D.M., Zhou S., Mak T.W., Hawke T.J. (2018). Loss of the adipokine lipocalin-2 impairs satellite cell activation and skeletal muscle regeneration. Am. J. Physiol. Cell Physiol..

[9] Ponzetti M., Aielli F., Ucci A., Cappariello A., Lombardi G., Teti A., Rucci N. (2021). Lipocalin 2 increases after high-intensity exercise in humans and influences muscle gene expression and differentiation in mice. J. Cell. Physiol..

[10] Marques F.Z., Prestes P.R., Byars S.G., Ritchie S.C., Würtz P., Patel S.K., Booth S.A., Rana I., Minoda Y., Berzins S.P. (2017). Experimental and Human Evidence for Lipocalin-2 (Neutrophil Gelatinase-Associated Lipocalin [NGAL]) in the Development of Cardiac Hypertrophy and heart failure. J. Am. Heart Assoc..

[11] Sung H.K., Chan Y.K., Han M., Jahng J.W.S., Song E., Danielson E., Berger T., Mak T.W., Sweeney G. (2017). Lipocalin-2 (NGAL) Attenuates Autophagy to Exacerbate Cardiac Apoptosis Induced by Myocardial Ischemia. J. Cell. Physiol..

[12] Xu G., Ahn J.H., Chang S.Y., Eguchi M., Ogier A., Han S.J., Park Y.S., Shim C.Y., Jang Y.S., Yang B. (2012). Lipocalin-2 induces cardiomyocyte apoptosis by increasing intracellular iron accumulation. J. Biol. Chem..

[13] Ding L., Hanawa H., Ota Y., Hasegawa G., Hao K., Asami F., Watanabe R., Yoshida T., Toba K., Yoshida K. (2010). Lipocalin-2/neutrophil gelatinase-B associated lipocalin is strongly induced in hearts of rats with autoimmune myocarditis and in human myocarditis. Circ. J..

[14] Moser H. (1984). Duchenne muscular dystrophy: Pathogenetic aspects and genetic prevention. Hum. Genet..

[15] Petrof B.J., Shrager J.B., Stedman H.H., Kelly A.M., Sweeney H.L. (1993). Dystrophin protects the sarcolemma from stresses developed during muscle contraction. Proc. Natl. Acad. Sci. USA.

[16] De Paepe B., De Bleecker J.L. (2013). Cytokines and chemokines as regulators of skeletal muscle inflammation: Presenting the case of Duchenne muscular dystrophy. Mediat. Inflamm..

[17] Birnkrant D.J., Bushby K., Bann C.M., Apkon S.D., Blackwell A., Brumbaugh D., Case L.E., Clemens P.R., Hadjiyannakis S., Pandya S. (2018). Diagnosis and management of Duchenne muscular dystrophy, part 1: Diagnosis, and neuromuscular, rehabilitation, endocrine, and gastrointestinal and nutritional management. Lancet Neurol..

[18] Bianchi M.L., Mazzanti A., Galbiati E., Saraifoger S., Dubini A., Cornelio F., Morandi L. (2003). Bone mineral density and bone metabolism in Duchenne muscular dystrophy. Osteoporos. Int..

[19] Takata S., Yasui N. (2001). Disuse osteoporosis. J. Med. Investig..

[20] Alexandre C., Vico L. (2011). Pathophysiology of bone loss in disuse osteoporosis. Jt. Bone Spine..

[21] Ginaldi L., Di Benedetto M.C., De Martinis M. (2005). Osteoporosis, inflammation and ageing. Immun. Ageing.

[22] Buckner J.L., Bowden S.A., Mahan J.D. (2015). Optimizing bone health in Duchenne muscular dystrophy. Int. J. Endocrinol..

[23] Rufo A., Del Fattore A., Capulli M., Carvello F., De Pasquale L., Ferrari S., Pierroz D., Morandi L., De Simone M., Rucci N. (2011). Mechanisms inducing low bone density in Duchenne muscular dystrophy in mice and humans. J. Bone Miner. Res..

[24] Birnkrant D.J., Bushby K., Bann C.M., Alman B.A., Apkon S.D., Blackwell A., Case L.E., Cripe L., Hadjiyannakis S., Olson A.K. (2018). Diagnosis and management of Duchenne muscular dystrophy, part 2: Respiratory, cardiac, bone health, and orthopaedic management. Lancet Neurol..

[25] De Almeida Hermes T., Mâncio R.D., Macedo A.B., Mizobuti D.S., Da Rocha G.L., Alves Cagnon V.H., Minatel E. (2019). Tempol treatment shows phenotype improvement in mdx mice. PLoS ONE.

[26] Yin H., Moulton H.M., Seow Y., Boyd C., Boutilier J., Iverson P., Wood M.J.A. (2008). Cell-penetrating peptide-conjugated antisense oligonucleotides restore systemic muscle and cardiac dystrophin expression and function. Hum. Mol. Genet..

[27] Capulli M., Rufo A., Teti A., Rucci N. (2009). Global transcriptome analysis in mouse calvarial osteoblasts highlights sets of genes regulated by modeled microgravity and identifies A “mechanoresponsive osteoblast gene signature”. J. Cell. Biochem..

[28] Abella V., Scotece M., Conde J., Gómez R., Lois A., Pino J., Gómez-Reino J.J., Lago F., Mobasheri A., Gualillo O. (2015). The potential of lipocalin-2/NGAL as biomarker for inflammatory and metabolic diseases. Biomarkers.

[29] Xiao X., Yeoh B.S., Vijay-Kumar M. (2017). Lipocalin 2: An Emerging Player in Iron Homeostasis and Inflammation. Annu. Rev. Nutr..

[30] Moschen A.R., Adolph T.E., Gerner R.R., Wieser V., Tilg H. (2017). Lipocalin-2: A Master Mediator of Intestinal and Metabolic Inflammation. Trends Endocrinol. Metab..

[31] Rosenberg A.S., Puig M., Nagaraju K., Hoffman E.P., Villalta S.A., Rao V.A., Wakefield L.M., Woodcock J. (2015). Immune-mediated pathology in Duchenne muscular dystrophy. Sci. Transl. Med..

[32] Conde J., Lazzaro V., Scotece M., Abella V., Villar R., López V., Gonzalez-Gay M., Pino J., Gómez R., Mera A. (2017). Corticoids synergize with IL-1 in the induction of LCN2. Osteoarthr. Cartil..

[33] Berger T., Togawa A., Duncan G.S., Elia A.J., You-Ten A., Wakeham A., Fong H.E.H., Cheung C.C., Mak T.W. (2006). Lipocalin 2-deficient mice exhibit increased sensitivity to Escherichia coli infection but not to ischemia-reperfusion injury. Proc. Natl. Acad. Sci. USA.

[34] Cheng L., Xing H., Mao X., Li L., Li X., Li Q. (2015). Lipocalin-2 promotes M1 macrophages polarization in a mouse cardiac ischaemia-reperfusion injury model. Scand. J. Immunol..

[35] Pelosi L., Berardinelli M.G., De Pasquale L., Nicoletti C., D’Amico A., Carvello F., Moneta G.M., Catizone A., Bertini E., De Benedetti F. (2015). Functional and Morphological Improvement of Dystrophic Muscle by Interleukin 6 Receptor Blockade. EBioMedicine.

[36] Gutpel K.M., Hrinivich W.T., Hoffman L.M. (2015). Skeletal muscle fibrosis in the mdx/utrn+/-mouse validates its suitability as a murine model of duchenne muscular dystrophy. PLoS ONE.

[37] Dempster D.W., Compston J.E., Drezner M.K., Glorieux F.H., Kanis J.A., Malluche H., Meunier P.J., Ott S.M., Recker R.R., Parfitt A.M. (2013). Standardized nomenclature, symbols, and units for bone histomorphometry: A 2012 update of the report of the ASBMR Histomorphometry Nomenclature Committee. J. Bone Miner. Res..

[38] Feldkamp L.A., Davis L.C., Kress J.W. (1984). Practical cone-beam algorithm. J. Opt. Soc. Am. A.

[39] Lorensen W.E., Cline H.E. (1987). Marching Cubes: A High Resolution 3D Surface Construction Algorithm. Comput. Graph..

[40] Vinet L., Zhedanov A. (2011). A “missing” family of classical orthogonal polynomials. J. Phys. A Math. Theor..

[41] Bouxsein M.L., Boyd S.K., Christiansen B.A., Guldberg R.E., Jepsen K.J., Müller R. (2010). Guidelines for assessment of bone microstructure in rodents using micro-computed tomography. J. Bone Miner. Res..

