# Supplementary material for: Lipocalin 2 Influences Bone and Muscle Phenotype in the MDX Mouse Model of Duchenne Muscular Dystrophy
Source: Int J Mol Sci. 2022 Jan 16;23(2):958. doi: 10.3390/ijms23020958 (PMC8780970; doi:10.3390/ijms23020958)
Supplement: Supplementary file 1 [file ijms-23-00958-s001.zip › Supplementary Figures DMD Ponzetti et al. revised IJMS.pptx]

## Slide 1
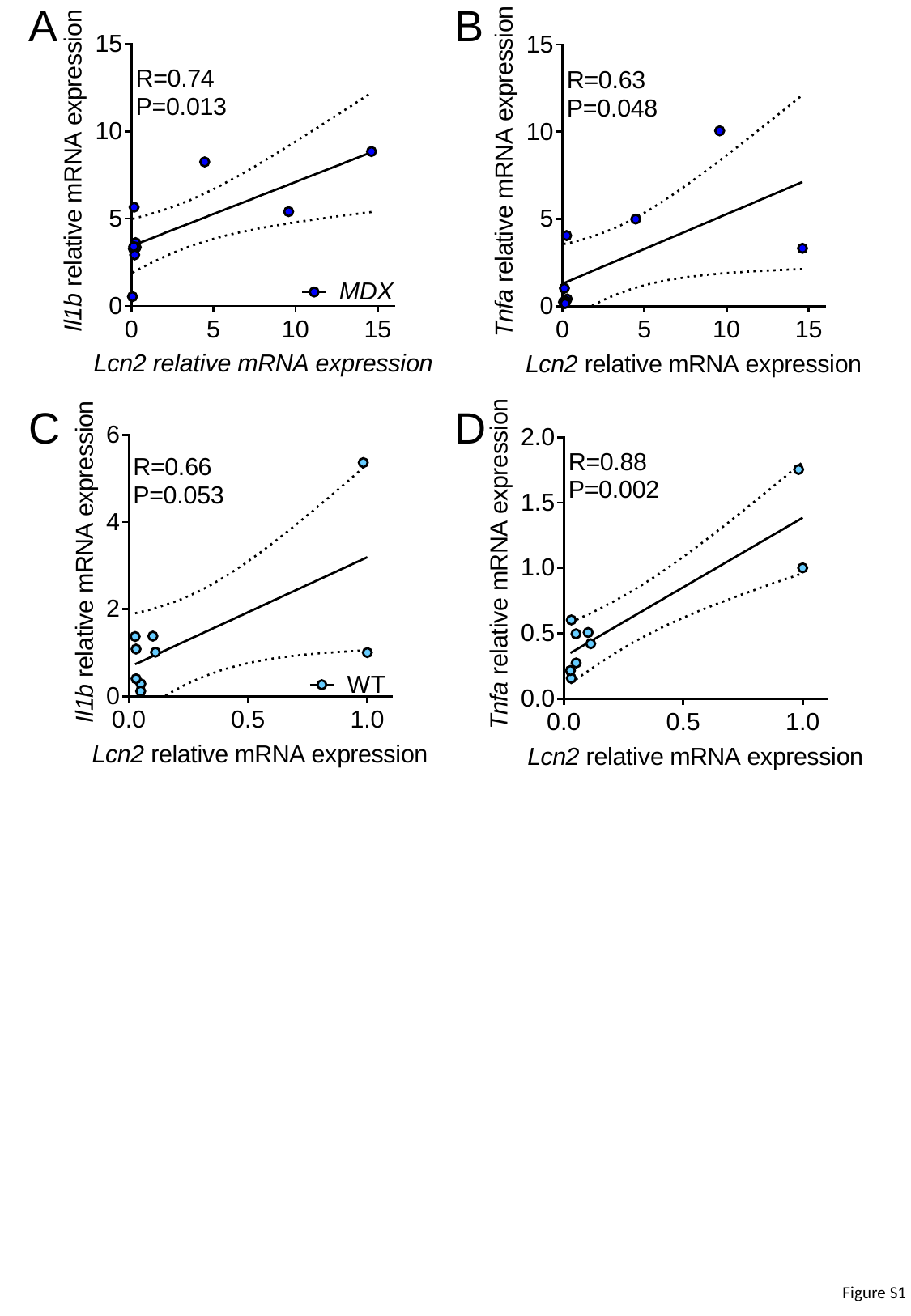

A
B
C
D
Figure S1

## Slide 2
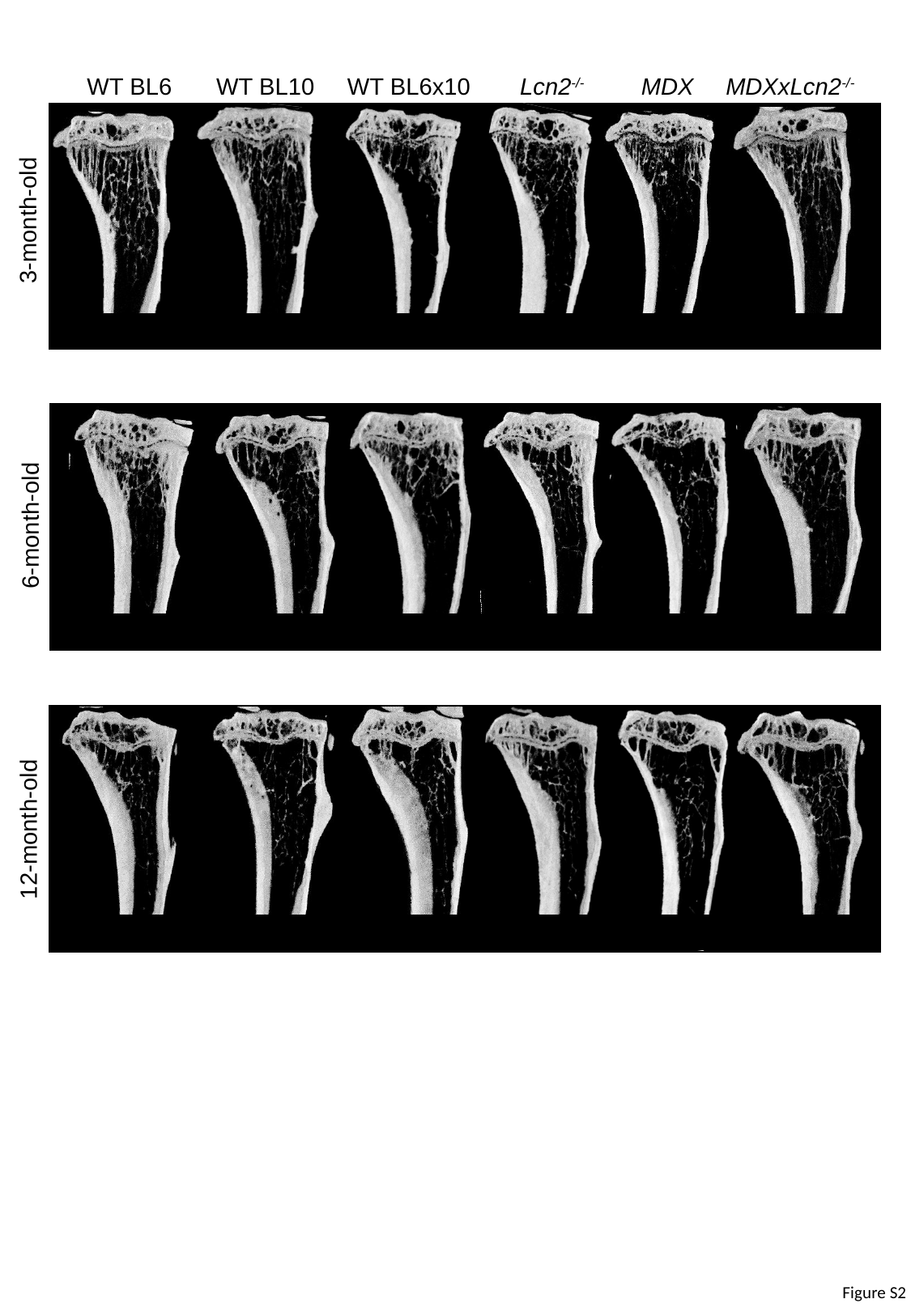

WT BL6
WT BL10
WT BL6x10
Lcn2-/-
MDX
MDXxLcn2-/-
3-month-old
6-month-old
12-month-old
Figure S2

## Slide 3
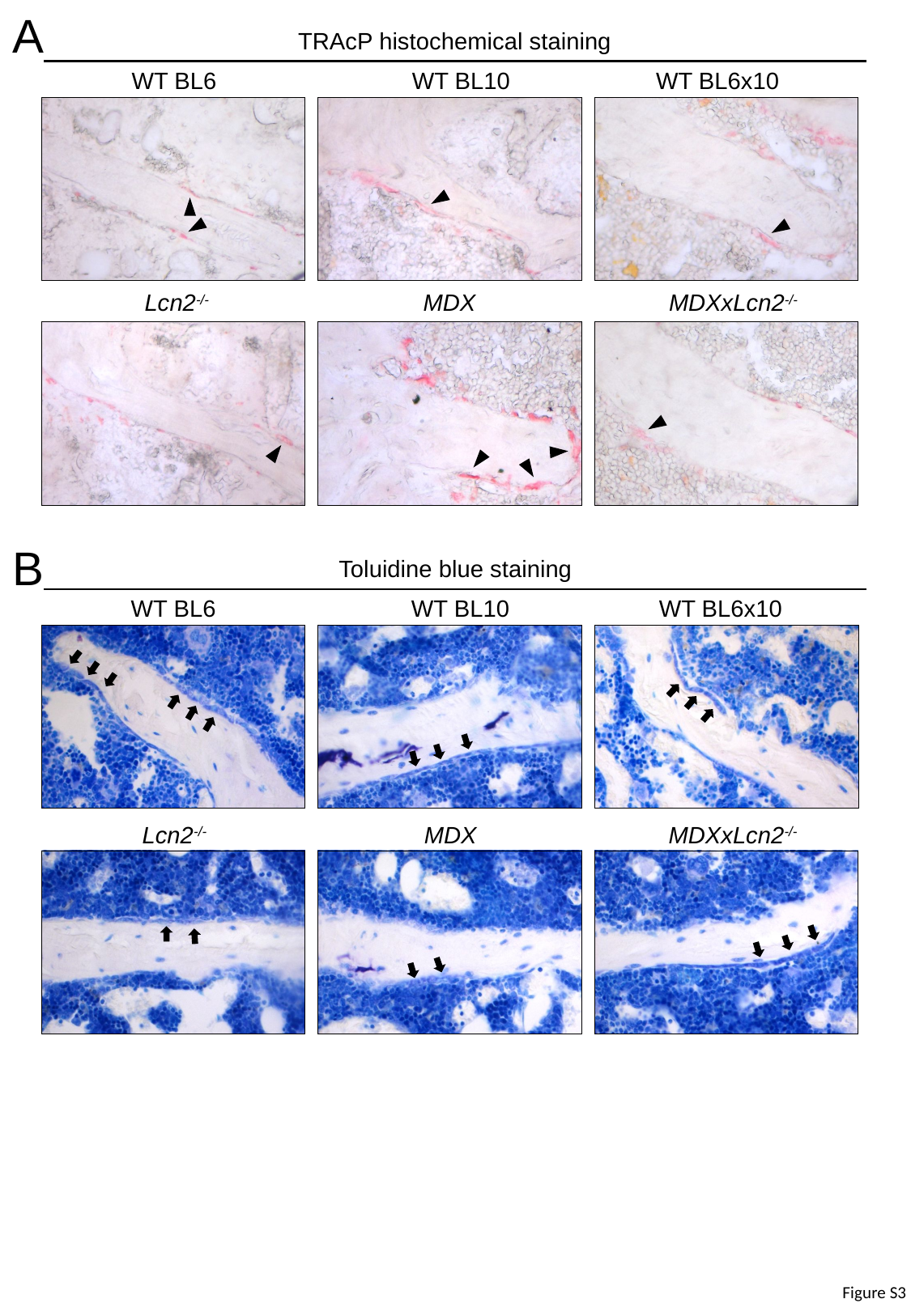

A
TRAcP histochemical staining
WT BL6
WT BL10
WT BL6x10
Lcn2-/-
MDX
MDXxLcn2-/-
B
Toluidine blue staining
WT BL6
WT BL10
WT BL6x10
Lcn2-/-
MDX
MDXxLcn2-/-
Figure S3

## Slide 4
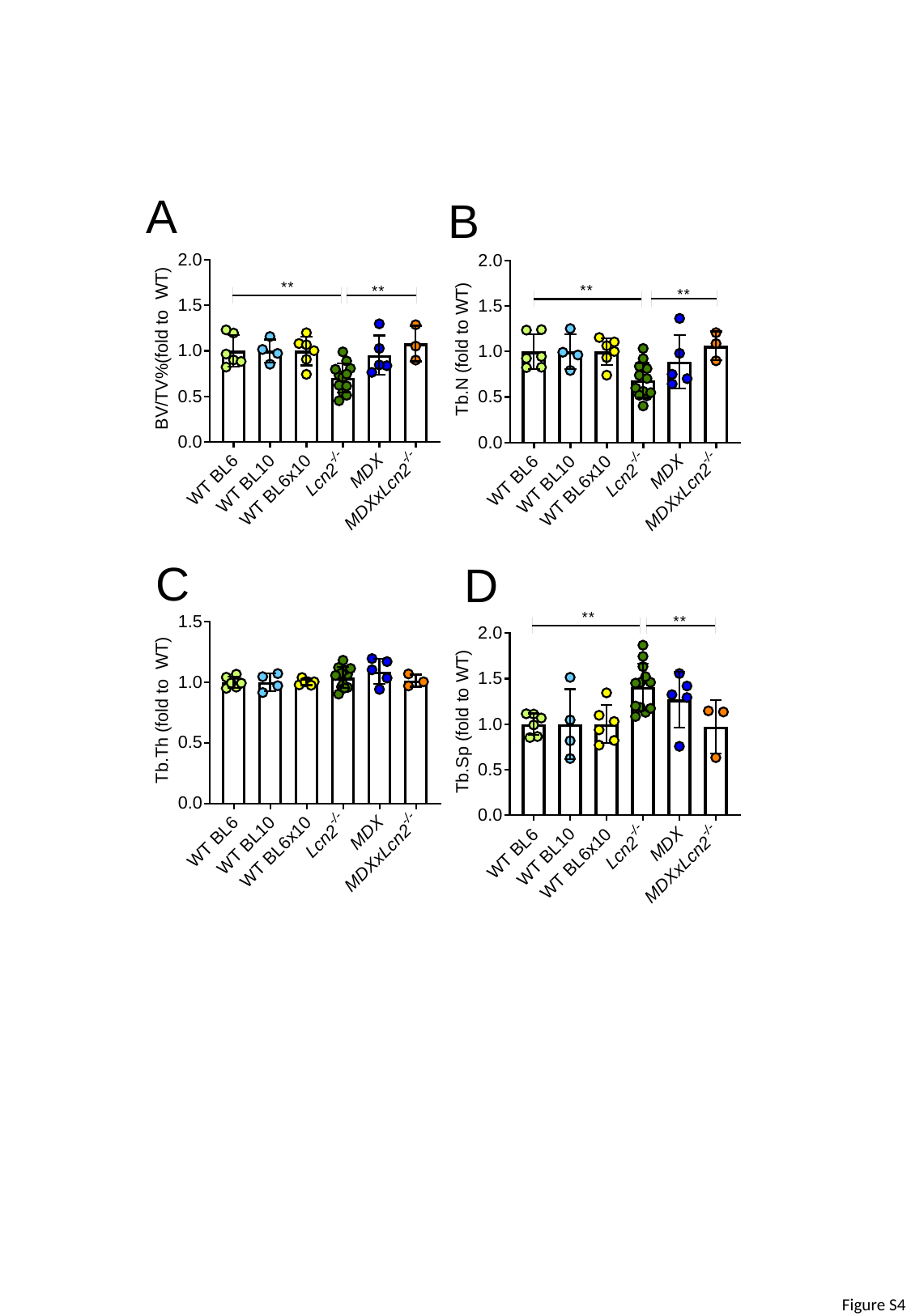

A
B
C
D
Figure S4

## Slide 5
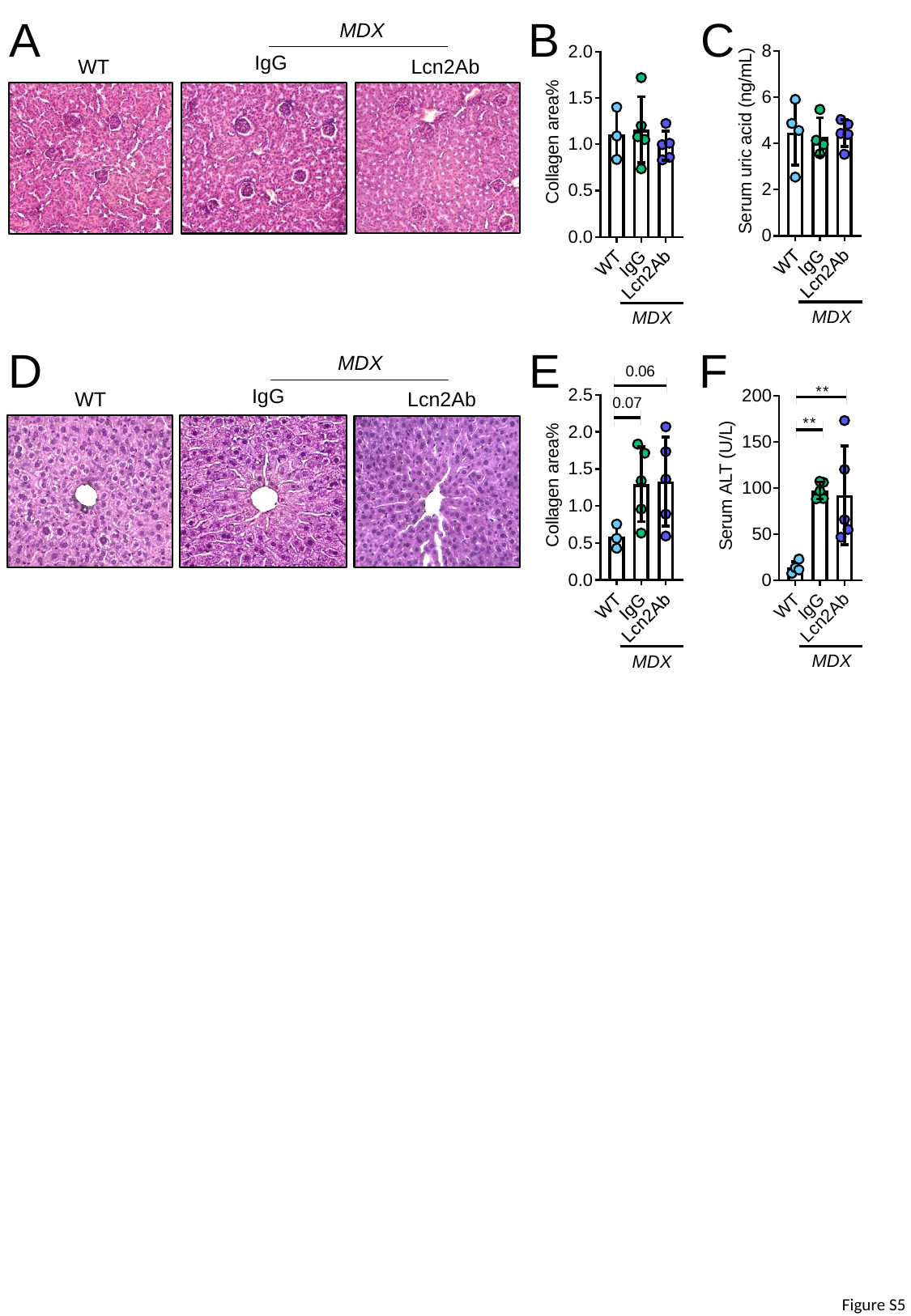

A
B
C
MDX
IgG
WT
Lcn2Ab
D
E
F
MDX
IgG
WT
Lcn2Ab
Figure S5
