# Supplementary material for: Lipocalin 2 Influences Bone and Muscle Phenotype in the MDX Mouse Model of Duchenne Muscular Dystrophy
Source: Int J Mol Sci. 2022 Jan 16;23(2):958. doi: 10.3390/ijms23020958 (PMC8780970; doi:10.3390/ijms23020958)
Supplement: Supplementary file 1 [file ijms-23-00958-s001.zip › Supplementary Figures legends Ponzetti et al. revised IJMS.pdf]

Supplementary Figures legends

Supplementary Figure S1: Correlation between Lcn2, *Il1b* and *Tnfa* mRNA expression. Correlation studies were ran on diaphragm, quadriceps, EDL and *soleus* muscles collected from *MDX* mice between the transcriptional expression of *Lcn2* and **(A)** *Il1b* or **(B)** *Tnfa*. The same analysis was performed between *Lcn2* and **(C)** *Il1b* or **(D)** *Tnfa* on muscles collected from WT mice. Pearson's correlation test.

Supplementary Figure S2: Evaluation of the bone phenotype by μCT. Representative 3D sections of tibiae explanted from 3-,6- and 12- month-old WT, *Lcn2*$^{-/-}$, *MDX* and *MDXxLcn2*$^{-/-}$ mice, fixed in 4% paraformaldehyde and subjected to μCT analysis.

Supplementary Figure S3: Evaluation of the bone phenotype by histomorphometry. Tibiae explanted from 6-month-old WT, *Lcn2*$^{-/-}$, *MDX* and *MDXxLcn2*$^{-/-}$ mice were fixed in 4% paraformaldehyde, embedded in poly-methyl-methacrylate, and sectioned at 5μm. Sections were then subjected to **(A)** TRAcP activity staining or **(B)** toluidine blue staining to perform histomorphometric analysis of osteoclast and osteoblast parameters, respectively. Arrowheads in **(A)** point at osteoclasts, arrows in **(B)** point at osteoblasts.

Supplementary Figure S4: Effect of *Lcn2* genetic ablation on bone phenotype of 12-month-old *MDX* mice. **(A)** MicroCT analysis performed on proximal tibiae explanted from 12-month-old *Lcn2*$^{-/-}$, *MDX* and *MDX* mice crossbred with *Lcn2*$^{-/-}$ (*MDX*x*Lcn2*$^{-/-}$) and their respective WTs (BL6, BL10, BL6x10 respectively) to evaluate trabecular bone volume/tissue volume (BV/TV%), **(B)** trabecular number (Tb.N), **(C)** thickness (Tb.Th) and **(D)** separation (Tb.Sp). *p<0.05, **p<0.01 and ***p<0.001 between the indicated groups. One-way *ANOVA*.

Supplementary Figure S5: Kidney and liver pathological examination after IgG or Lcn2Ab curative treatment. Kidney and liver were explanted from mice treated with IgG or Lcn2Ab (see main figure 6A), fixed in 4% paraformaldehyde, embedded in paraffin, sectioned at 5μm and subjected to **(A,D)** haematoxylin/eosin staining to evaluate histological appearance. **(B,E)** Masson's Trichrome staining to assess **(B)** kidney and **(E)** liver fibrosis. **(C,F)** ELISA assay to evaluate serum levels of **(C)** the kidney damage marker urea, and **(F)** the liver damage marker alanine transaminase (AST). One-way *ANOVA*. **p<0.01 between the indicated groups.
