# Supplementary material for: Lipocalin 2 Influences Bone and Muscle Phenotype in the MDX Mouse Model of Duchenne Muscular Dystrophy
Source: Int J Mol Sci. 2022 Jan 16;23(2):958. doi: 10.3390/ijms23020958 (PMC8780970; doi:10.3390/ijms23020958)
Supplement: Supplementary file 1 [file ijms-23-00958-s001.zip › Supplementary tables Ponzetti et al. revised IJMS.pdf]

Supplementary Table SI: Sequences of PCR primers used in this study

**Gene**	**Forward primer**	**Reverse primer**
Il6	GAGGATACCACTCCCAACAGACC	AAGTGCATCATCGTTGTTCATACA
Il1b	GCCCATCCTCTGTGACTCAT	AGGCCACAGGTATTTTGTCG
Tnfa	CTCCCTTTGCAGAACTCAGG	AGCCCCCAGTCTGTATCCTT
Myod1	TACAGTGGCGACTCAGATGC	TAGTAGGCGGTGTCGTAGCC
Myog	CCAACCCAGGAGATCATTTG	ATATCCTCCACCGTGATGCT
Pax7	TGGCCAAACTGCTGTTGATTAC	CGTCTCCAGAGGGTTTCCTG
Col1a1	GTCCCTCTGGAAATGCTGGAC	GACCGGGAAGACCGACCA
Col3a1	GACCAGCAGTCCAACGTAGA	TCTCCAAATGGGATCTGTGG
Gapdh	TGTGAGGGAGATGCTCAGTG	TGTTCCTACCCCCAATGTGT

Supplementary Table SII: effect of Lcn2 genetic ablation on bone phenotype of 3-month-old male *MDX* mice. Values are shown as Mean±SD of the non-normalized values. Statistics were performed by One-way ANOVA.

	WT BL6	WT BL10	WT BL6x10	$Lcn2^{-/-}$	*MDX*	$MDXxLcn2^{-/-}$
BV/TV%	17.91±2.613	16.03±3.706	13.76±1.597$^{\$}$	13.56±1.245$^{\$}$	9.949±0.8516†	14.63±4.589*
Tb.N (1/mm)	4.567±0.430	4.515±1.107	3.843±0.3968	3.699±0.373$^{\$}$	2.85±0.2974†	3.91±1.151*
Tb.Th (µm)	39.18±3.874	35.66±2.072$^{\$}$	35.81±2.358$^{\$}$	36.72±1.46$^{\$}$	35.3±5.383	37.33±3.164
Tb.Sp (µm)	101.0±11.3	112.1±22.01	111±17.69	134.5±19.44$^{\$}$	153±30.87†	109.7±39.25*
Ct.Th (µm)	90.85±4.857	86.95±6.467	83.66±14.29	91.38±14.24	88.68±18.29	91.46±9.035
Serum CTx (ng/ml)	13.97±3.964	6.132±7.058	15.57±6.321†	10.33±8.554	18.77±10.92†	17.71±13.48
Oc.N/BS (1/mm)	2.594±0.4183	2.197±0.2826	2.548±0.669	2.284±0.491	3.414±0.7533†	1.236±0.557$^{¥\ddagger *}$
Oc.S/BS%	7.354±1.299	5.399±0.7867	6.982±1.466	7.850±3.162	9.204±2.383†	4.987±1.241$^{\ddagger *}$
Ob.N/BS (1/mm)	6.52±1.833	4.81±1.383	5.826±1.681	2.828±0.277$^{\$}$	3.105±0.68	5.564±1.870$^{\ddagger *}$
Ob.S/BS%	11.08±2.533	8.303±2.435	7.418±1.019$^{\$}$	4.704±0.738$^{\$}$	5.620±1.024	8.636±2.607$^{\ddagger *}$
ID (µm)	37.67±5.538	35±5.568	32.63±2.56	43.5±8.093	40.25±5.62	34.67±1.751‡
TID (µm)	40.83±5.529	36±6.928	35.13±3.044	46.67±8.262	43.75±6.702	37.33±2.503‡

p≤0.05 *vs* $^{\$}$WT BL6; †WT BL10; $^{¥}$WT BL6x10; **MDX*; $^{\ddagger}Lcn2^{-/-}$

Supplementary Table SIII: effect of Lcn2 genetic ablation on bone phenotype of 6-month-old male *MDX* mice. Values are shown as Mean±SD of the non-normalized values. Statistics were performed by One-way ANOVA.

	WT BL6	WT BL10	WT BL6x10	$Lcn2^{-/-}$	*MDX*	$MDXxLcn2^{-/-}$
BV/TV%	15.6±2.931	10.85±0.849$^{\$}$	10.23±1.439$^{\$}$	8.37±2.919$^{\$}$	7.974±1.264†	11.24±2.62*
Tb.N (1/mm)	3.645±0.4089	2.52±0.448$^{\$}$	2.636±0.332$^{\$}$	2.208±0.618$^{\$}$	2.15±0.4369	3.003±0.5928$^{\ddagger *}$
Tb.Th (µm)	42.52±3.382	40.39±4.026	39.54±2.33	37.49±4.14$^{\$}$	36.42±4.309†	37.16±2.73
Tb.Sp (µm)	154.9±10.82	136.2±40.63	141±20.59	187.9±19.86	158.3±14.69†	158.3±14.69*
Ct.Th (µm)	121.8±13.16	115±8.96	105±11.54$^{\$}$	120.2±11.35	93.34±15.69†	115.1±14.86*
Oc.N/BS (1/mm)	1.976±0.437	1.612±0.1166	2.337±0.7568	2.042±0.294	2.622±0.8622†	1.732±0.5207*
Oc.S/BS%	7.646±1.744	7.358±0.8302	7.18±0.7617	7.757±1.224	10.92±2.543†	7.665±2.2*
Ob.N/BS (1/mm)	21.03±3.829	13.41±2.628$^{\$}$	14.44±3.891$^{\$}$	10.73±0.826$^{\$}$	11.52±1.884	16.1±3.373$^{\ddagger *}$

Ob.S/BS%	15.06±1.228	11.84±1.161	11.94±0.9753	9.576±2.149$^{\$}$	9.48±2.718	15.68±5.076‡*
ID (µm)	32.33±3.428	34.67±6.028	33.13±7.53	36.67±7.607	45±13.54	32.4±5.595*
TID (µm)	36.22±4.41	39.33±6.658	37.25±8.242	40.33±8.847	49.5±14.75	35.4±5.983*

p≤0.05 *vs* $^{\$}$WT BL6; †WT BL10; **MDX*; ‡*Lcn2*$^{-/-}$

Supplementary Table SIV: effect of Lcn2 genetic ablation on bone phenotype of 12-month-old male *MDX* mice. Values are shown as Mean±SD of the non-normalized values. Statistics were performed by One-way ANOVA.

	WT BL6	WT BL10	WT BL6x10	*Lcn2*$^{-/-}$	*MDX*	*MDXxLcn2*$^{-/-}$
BV/TV%	9.296±1.598	4.683±0.587$^{\$}$	5.658±0.897$^{\$}$	6.526±1.474$^{\$}$	4.47±1.01	6.112±1.108
Tb.N (1/mm)	2.378±0.5898	1.62±0.6889	1.96±0.4327	2.028±0.7454	2.103±0.6276	2.78±0.2762$^{¥*}$
Tb.Th (µm)	27.58±1.257	27.98±2.00	27.22±0.626	28.65±2.357	30.44±2.891	27.58±1.358
Tb.Sp (µm)	141.3±16.69	200.2±76.98	197.1±41.14$^{\$}$	199±36.3$^{\$}$	254.1±61.08	191.2±57.77
Serum CK (U/L)	911.4±1134	615±512.5	329±185.3	768.4±679.2	2709±3435	3624±3309$^{¥‡}$

p≤0.05 *vs* $^{\$}$WT BL6; $^{¥}$WT BL6x10; **MDX*; ‡*Lcn2*$^{-/-}$

Supplementary Table SV: effect of Lcn2 genetic ablation on muscle phenotype of male *MDX* mice. Values are shown as Mean±SD of the non-normalized values. Statistics were performed by One-way ANOVA.

	WT BL6	WT BL10	WT BL6x10	*Lcn2*$^{-/-}$	*MDX*	*MDXxLcn2*$^{-/-}$
				3-month-old		
Grip force(fold to BW)	3.81±0.6742	3.544±0.335	3.447±0.44$^{\$}$	3.467±0.5709	2.411±0.3705†	3.295±0.478*
Grip force (g)	102.9±19.81	92.26±8.38	87.22±16.76	87.27±12.54$^{\$}$	70.69±11.21†	91.01±14.17*
Intact fibres%	99.21±0.6991	98.53±2.019	97.95±1.593	99.39±0.0836	17.3±6.85†	20.37±6.29$^{¥‡}$
Collagen area%	0.7544±0.308	1.157±0.2857	1.036±0.4738	0.9958±0.694	4.859±1.359†	2.554±1.289$^{¥‡*}$
Serum CK (U/L)	427.4±311.3	456.8±363.2	1358±1494	712.8±620	20586±12255†	5966±5406‡*
				6-month-old		
Grip force(fold to BW)	3.023±0.4854	2.825±0.4315	2.823±0.5259	3.18±0.5516	2.041±0.466†	2.526±0.455‡*
Grip force (g)	82.62±15.12	87.15±13.57	89.95±19.42	95.77±15.71$^{\$}$	65.51±15.65†	79.33±14.51‡
Intact fibres%	97.81±1.487	95.53±1.416	94.82±0.569$^{\$}$	98.55±0.5256	26.9±4.081†	30.51±8.72$^{¥‡}$
Collagen area%	2.966±1.379	2.724±1.318	2.116±0.881	3.926±0.3995	6.864±0.4363†	4.229±1.175$^{¥*}$
Serum CK (U/L)	1146±1433	949.9±1063	1235±483.9	650.3±1031	2258±1648†	1378±1437

p≤0.05 *vs* $^{\$}$WT BL6; †WT BL10; $^{¥}$WT BL6x10; **MDX*; ‡*Lcn2*$^{-/-}$
